# Intravenous Drug Users Can Achieve a High Sustained Virological Response Rate: experience From Croatian Reference Center for Viral Hepatitis

**DOI:** 10.5812/kowsar.1735143x.4216

**Published:** 2011-12-20

**Authors:** Ivan Kurelac, Neven Papic, Slavko Sakoman, Mirjana Orban, Davorka Dusek, Marijana Coric, Adriana Vince

**Affiliations:** 1 Croatian Reference Center for Viral Hepatitis, University Hospital for Infectious Diseases, Zagreb, Croatia; 2 Department of psychiatry, Ward of Addictions, Sisters of Mercy University Hospital, Zagreb, Croatia; 3 Department of Pathology, Clinical Hospital Centre Zagreb, Zagreb, Croatia

**Keywords:** Behavior, Addictive, Injections, Infusions, Intravenous, Hepatitis, Chronic, Hepatitis C

## Abstract

**Background:**

Hepatitis C virus (HCV) is one of the major infectious disease agents among injecting drug users (IVDUs). However, most of the IVDUs are not still treated.

**Objectives:**

To examine the treatment course, adherence, tolerability and safety profiles and SVR rates in IVDUs compared to non-IVDUs.

**Patients and Methods:**

Demographic and clinical data were collected from medical records of 345 adult patients diagnosed with chronic hepatitis C (CHC) who were treated with a PEG-IFN-α and ribavirin in Croatian Reference Center for Viral Hepatitis in Zagreb between January 2003 and January 2010. Efficacy, safety and tolerability treatment profiles were analyzed in IVDUs vs. non-IVDUs. Positive predictors for treatment outcome were evaluated by univariate and multivariate logistic regression.

**Results:**

A total of 106 (30.46%) IVDUs were identified. The IVDUs were mainly male (81.13% vs. 52.30%, P = 0.0001), young (mean ± SD age: 32.46 ± 5.33 y vs. 46.12 ± 11.48 y, P = 0.0001), had lower fibrosis and HAI score (measured by ISHAK) and shorter duration of infection (mean ± SD: 8.98 ± 5.87 vs. 16.79 ± 8.99 y, P = 0.0001) compared to non-IVDU group. In IVDUs, genotype 1a (24.52%) and 3a (38.68%) were predominant. There were no differences in completion rate between the two studied groups. IVDUs achieved a significantly higher rate of overall SVR (70.75% vs. 51.04%, P < 0.0009) and in genotypes 1 and 4 (65.08% vs. 48.73%, P = 0.0294) vs. non-IVDUs. Treatment discontinuation rates due to side-effects were not significantly different in IVDUs and non-IVDUs (2.83% vs. 7.11%, P = 0.1390). IVDU group had a higher rate of lost to follow-up (13.21% vs. 4.60%, P = 0.0071). There were no statistically significant differences in SVR rate between IVDUs with, or without substitution therapy (55.55% vs. 74.62%, P = 0.0866). Independent predictors of SVR were age < 40 years and genotypes 2 and 3. Type of PEG-IFN-α used was not associated with SVR.

**Conclusions:**

Treatment of CHC in IVDUs should strongly be encouraged as they have positive predictors for achieving SVR such as younger age, shorter duration of infection, and consequently favorable histological stage of the disease, and good adherence to treatment. There is no difference in safety and tolerability profiles of treatment in IVDUs compared to patients with no history of drug abuse.

## 1. Background

The disordered life-style of many intravenous drug users (IVDUs) represents a serious health risk and is strongly associated with social disadvantages. Sharing equipment for drug injection is the most important factor for blood-borne infection transmission [1]. IVDU is associated with high risk for transmission of human immunodeficiency virus (HIV) and hepatitis B (HBV) and C (HCV). HCV is far more infectious than HIV in terms of blood-borne transmission, and shows much higher seroprevalence rates among IVDUs [1][2][3]. Studies show that 60%-90% of patients with intravenous drug abuse are chronically infected with HCV. Incoming data reveals a prevalence of HCV among new IVDUs (injecting less than two years) of 40%, making this problem a global public health priority [4][5][6]. Currently available standard-of-care, pegylated interferon-alpha (PEG-IFN-α) and ribavirin (RBV) therapy, successfully eradicates HCV infection in up to 55% of patients, as measured by sustained virological response (SVR) [7][8]. However, this treatment has numerous side effects, among which neuropsychiatric disorders play an important role in keeping adherence to the therapy [7][8].

Previous treatment guidelines and recommendations for the management of chronic hepatitis C (CHC) discouraged treatment of HCV in IVDUs [9], but even after the guidelines have been liberalized over the past ten years, most of IVUDs are still not treated [10]. Concerns about the abstinence, risk of reinfection, concomitant substitution therapy and alcohol abuse, and low treatment compliance, are the main reasons for treatment denial [10][11]. Only one-third of the infected former IVDUs receive antiviral treatment [10][12]. However, recently published studies contradict this prejudice by showing that IVDUs can be treated effectively and safely, especially those on methadone maintenance or similar opiate substitution therapy [13][14][15]. Moreover, treatment of IVDUs becomes imperative for every efficient public health program. There can be no decrease in HCV prevalence in general population without management of infection in IVDUs. The reported prevalence rate of IVDU in European Union is estimated to be between 3.6 and 4.4 cases per 1000 (in population aged 15-64 years), and the general decreasing trend has been noticed over the past ten years [4]. Reports from Croatia show similar prevalence and stable epidemiological situation. Similar to some other European countries, Croatia has high HCV prevalence among IVDUs [4][16][17][18]. Kolaric, et al., reported the seroprevalence of anti-HCV up to 65%, among imprisoned IVDUs in the three largest Croatian cities, representing a significant burden to Croatian health care system [16].

## 2. Objectives

This study was conducted to examine the treatment course, safety and tolerability profile, adherence to treatment and rates of SVR in IVDUs compared to non-IVDUs with CHC, treated at the Croatian Reference Center for Viral Hepatitis, the largest center for HCV-positive patients in Croatia.

## 3. Patients and Methods

A retrospective study including 345 adult patients, 211 men (61.16%) and 134 women (38.84%), with a mean ± SD age of 41.92 ± 11.82 years, was conducted at the Croatian Reference Center for Viral Hepatitis, Zagreb, Croatia. A total of 106 IVDUs were recognized in the group, representing 30.46% of those treated. A history of blood transfusion was a clear risk factor for HCV in 112 (32.46%) patients. A history of having been stuck with a bloody needle or sharp instrument (not IVDU-associated) was reported in 49 (14.2%) patients, sexual-behavior risk factors in 7 (2.03%) patients and the risk associated with tattooing was reported in 1 (0.31%) patient. In 70 (20.28%) patients risk factor remained unknown.

Two-hundred and one (58.26%) patients were treated with Peg-IFN-α2a 180 μg/week + RBV; 144 (41.74%) were treated with Peg-IFN-α2b 1.5 μg/week + RBV. RBV was administered at the recommended dosage. The duration of therapy in genotypes 1 and 4 was 48 weeks, and in genotypes 2 and 3 was 24 weeks. Therapy was discontinued in HCV genotype 1 and HCV genotype 4 patients if viral load decreased by less than 2 log HCV RNA copies/mL at week 12 compared with baseline values or if HCV RNA was still detectable at week 24. In this study, we compared the age, gender, genotype, stage of fibrosis, type of PEG-IFN-α, treatment safety profile and the sustained virological response (SVR) rates among IVDUs and patients with no history of drug use. The second objective of this study was to assess the influence of substitution therapy on achieving SVR in the group of IVDUs.

### 3.1. Definitions

SVR was defined as undetectable HCV RNA 24 weeks after therapy with combined PEG-IFNα-2a or α-2b plus RBV. HCV RNA quantification was performed by using COBAS Ampliprep/COBAS TaqMan HCV test (Roche diagnostics, diagnostic systems Pleasanton, CA). HCV genotyping was performed by using VERSANT HCV Genotyping assay (LIPA) (Bayer diagnostics, Puteaux, Cedex, France) at the Department of Molecular Diagnostics and Cellular Immunity, UHID. Liver biopsy was performed at the Department of Viral Hepatitis, UHID and the ISHAK scoring system was used to assess the histological activity [19]. Disease duration was estimated by considering the date of blood transfusion received prior to 1993, the date of surgery, or the period of drug injection as the onset of infection. All the IVDUs treated at UHID were abstinent for at least six months, so the active drug users were not enrolled in our study.

### 3.2. Data Collection

Patient records of all patients that started treatment at UHID between January 2003 and January 2010 were extracted and used for collection of clinical and laboratory data. Ethics Committee of UHID approved this study. Virologic results and liver biopsy data were double-checked at the available databases of the Department of Molecular Diagnostic and Cellular Immunity, UHID, and Department for Viral Hepatitis, UHID.

### 3.3. Statistical Analysis

Statistical analyses were performed using Prism (ver 5.0) statistical software (GraphPad Software, San Diego, CA) and MedCalc for Windows®, ver 11.5.1.0 (MedCalc Software, Mariakerke, Belgium). The demographic, clinical characteristics and laboratory data were evaluated and presented descriptively. χ2, Fisher's exact test and Mann Whitney U test were used to compare the groups, as appropriate. All tests were two-tailed; a P < 0.05 was considered statistically significant. Binary logistic regression analysis was used to assess the independent predictors of SVR. The strength of association was expressed as odds ratio (OR) and its corresponding 95% confidence interval (CI). The unadjusted univariate OR was also calculated for every predictor. All predictors were entered in a backward stepwise logistic regression model with SVR being the dependent variable. The initial model included all predictors: antiviral therapy (Peg-IFN-α2a vs. Peg-IFN-α2b), age (≤ 40 years vs. > 40 years), gender, body weight (≤ 75 kg vs. > 75 kg), HCV-genotype (1 and 4 vs. 2 and 3), extent of fibrosis (mild fibrosis: ISHAK 0-2 vs. significant fibrosis: ISHAK 3-6), presence of risk factor (IVDU vs. non-IVDU), and duration of infection. Statistically non-significant predictors were progressively excluded based on a likelihood ratio test.

## 4. Results

### 4.1. Patients Characteristics

The IVDUs group was predominately young males with lower extent of fibrosis and HAI score (measured by ISHAK) and had typically a shorter duration of infection than non-IVDU group. There were significant differences in genotype distribution between the two groups: The genotype 1b was strongly associated with patients without history of drug abuse (transfusion and surgery), while in IVUDs genotypes 1a and 3a were predominant, with 3a being far more frequent than in other populations. The mean ± SD duration of IVDU abstinence was 4.76 ± 3.16 (range: 0.5-18) years. The distribution of selected variables is presented in Table 1.

**Table 1:** Baseline Characteristics of the patients With Chronic Hepatitis C enrolled in the Study

	**Intravenous Drug Users (n = 106)**	**No History of Drug Abuse (n = 239)**	**P value**
Demographic characteristics, No. (%)			
Male	86 (81.13)	125 (52.30)	0.0001^b^
Female	20 (18.87)	114 (47.70)	
ge, y, mean ± SD a	32.46 ± 5.33	46.12 ± 11.48	0.0001^c^
Weight, kg, mean ± SD	78.94 ± 15.02	73.57 ± 14.27	0.0129^c^
Genotype, No. (%)			
Total d	59 (55.66)	188 (78.66)	0.0001^b^
Subtype 1a	26 (24.52)	15 (6.27)	0.0001^b^
Subtype 1b	20 (18.87)	129 (53.97)	0.0001^b^
Subtype 2	2 (1.87)	3 (1.25)	0.6448^b^
Subtype 3/3 a	41 (38.68)	39 (16.32)	0.0001^b^
Subtype 4	4 (3.79)	9 (3.77)	0.5034^b^
Liver biopsy (ISHAK score) (n = 262, 81+181)			
Fibrosis; median (IQR a)	3 (3-3)	3 (3-4)	0.004 c
Histology activity score; median (IQR)	7 (6-9)	8 (6-11)	0.008 c
Duration of infection, y, mean ± SD	8.98 ± 5.87	16.79 ± 8.99	0.001 c
Therapy, No. (%) (n = 189, 83+106)			
PEG-IFN-α2a a	67 (63.20)	134 (56.07)	0.1917^b^
PEG-IFN-α2b	39 (36.80)	105 (43.93)	

^a^ Abbreviations: IQR, interquartile range; peG-IFn-α, pegylated interferon alpha; SD, Standard deviation

^b^ Fisher's exact test

^c^ Mann-Whitney U test

^d^ In 57 patients genotype 1 was not typeable

### 4.2. Treatment Safety, Modifications and Discontinuation

The safety and tolerability profiles of treatment were compared in IVDU and non-IVDU groups. Both groups had similar frequencies of side-effects (despite the different response rates described below). Among IVDU patients, 75 (70.75%) reported at least one side-effect, but only 12 of them (11.32%) required dose reduction and 3 (2.83%) needed therapy discontinuation. Sixteen (15.09%) patients reported only one side-effect, 35 (33.33%) two, and 24 (22.64%) three or more side-effects. Among patients with no history of drug abuse, 178 (74.47%) reported side-effects, 38 (15.89%) required dose reduction and 17 (7.11%) needed therapy discontinuation. Twenty-six (10.87%) patients reported one, 64 (26.78%) two and 88 (36.82%) three or more side-effects. The frequency of most common side-effects is shown in Table 2. Next, we analyzed possible safety differences IVDU patients treated with two different PEG-IFN-α regiments. Regarding psychiatric side-effects, it was recorded that anxiety, depression and insomnia were more frequent in IVDU group, although it did not reach a statistically significant level (Table 2)

**Table 2:** Reported Side-effects During peG-IFn-α Therapy

	**Intravenous Drug Users, No. (%) (n = 106)**	**No History of Drug Abuse, No. (%)(n = 239)**
Anemia	19 (17.92)	45 (18.82)
Neutropenia	7 (6.66)	26 (10.87)
Thrombocytopenia	5 (4.71)	19 (7.94)
Atralgiae/mialgiae	8 (7.54)	21 (8.78)
Dermatological (including dermatitis, rash, pruritus)	26 (24.52)	68 (28.45)
Hipo/hiperthyreosis	3 (2.83)	6 (2.51)
Flu-like symptoms	49 (46.23)	108 (45.18)
Respiratory symptoms	2 (1.88)	3 (1.25)
Anxiety	21 (19.81)	35 (14.64)
Depression	9 (8.49)	6 (2.51)
Insomnia	8 (7.54)	6 (2.51)
Headache	36 (33.96)	78 (32.63)

Psychiatric side-effects were more frequent in patients treated with PEG-IFN-α2a, but there were no statistically significant differences in general and psychiatric side-effects. Among IVDUs treated with PEG-IFN-2α, 14 (20.89%) patients reported anxiety problems, 6 (8.95%) insomnia and 8 (11.94%) depression. In IVDUs treated with PEG-IFN-2αb, 7 (17.94%) reported anxiety problems, 2 (5.13%) insomnia and one depression (2.56%). Moreover, correlation analysis did not reveal any association between the type of PEG-IFN administered and therapy modification or discontinuation (OR = 1.87; 95% CI: 0.74-4.42; P = 0.1441). However, the influence of relatively small sample of patients within analyzed variables cannot be excluded.

Analysis of the reduction of therapy was evaluated in all 345 patients (Table 3). There were no statistically significant differences in reduction and discontinuation rates between the two groups. The reasons for treatment discontinuation in IVDU group were severe anemia, neutropenia and severe depression that did not improve after dose reduction and supportive therapy.

### 4.3. Virological Response

Analysis included data from all patients who received at least one dose of medication (intention-to-treat analysis). In the whole group, SVR was achieved in 197 (57.10%) of 345 patients, SVR rate for genotypes 1 and 4 was 52.69% and for genotypes 2 and 3 was 74.11% (Table 3).

**Table 3:** Treatment Modification and Sustained Virological Response (SVR)

	**Intravenous Drug Users, No. (%)**	**No History of Drug Abuse, No. (%)**	**P values b**
SVR a all genotypes	75 (70.75)	122 (51.04)	0.0009
Genotypes 1 and 4	41 (65.08)	96 (48, 73)	0.0294
PEG-IFN-α2a +RBV a	26 (66.66)	-	
PEG-IFN-α2b +RBV	15 (62.50)	-	
PEG-IFN-α2a +RBV	-	56 (49.57)	
PEG-IFN-α2b +RBV	-	38 (44.18)	
Genotypes 2 and 3	34 (79.07)	29 (69.05)	0.3300
PEG-IFN-α2a +RBV	21 (75.00)	-	
PEG-IFN-α2b +RBV	13 (86.66)	-	
PEG-IFN-α2a +RBV	-	13 (61.69)	
PEG-IFN-α2b +RBV	-	15 (75.00)	
Lost of follow-up	14 (13.21)	11 (4.60)	0.0071
Therapy modification			
Reduction of drug doses	12 (11.32) c	38 (15.89) d	0.3210
PEG-IFN-α a dose reduction	6 (5.66)	14 (5,86)	1.0000
RBV dose reduction	4 (3.77)	19 (7.95)	0.1694
PEG-IFN-α and RBV dose reduction	2 (1.89)	5 (2.09)	1.0000
Therapy discontinuation	3 (2.83)	17 (7.11)	0.1390

^a^ Abbreviations: peG-IFn-α, pegylated-interferon alpha; RBV, ribavirin; SVR, sustained virological response

^b^ Fisher's exact test

^c^ Reasons for treatment modification in IVDUs: anemia 4, neutropenia 3, hypothyroidism 2, depression 2, pancytopenia 1

^d^ Reasons for treatment modification: anemia 14, neutropenia 8, thrombocytopenia 7, depression 5, hypothyroidism 4

To determine influence of previous intravenous drug use on achieving SVR, IVDUs and non-IVDUs groups were compared. Overall, IVDUs group had a higher SVR rate (70.75% vs. 51.04%, P = 0.0009), particularly in those with genotypes 1 and 4 (65.08% vs. 48.73%, P = 0.0294, Fisher's exact test) when compared to non-IVDU group. Although the SVR rate was better in IVDU patients with genotypes 2 and 3 than in non-IVDU patients, the difference was not statistically significant. Additionally, IVDU group had a higher rate of lost to follow-up (13.21% vs. 4.60%, P = 0.0071), i.e. 13% of IVDUs did not come for final evaluation 24 weeks after therapy. There was no statistically significant difference in SVR rates between patients treated with PEG-IFN-α2a and PEG-IFN-α2b.

### 4.4. Influence of Substitution Therapy With Methadone/Buprenorphine on Adherence and SVR

Data for substitution therapy were available for 94 (88.68%) of 106 patients. There were no complete medical records for 12 patients. According to records, 12 (12.76%) patients were on methadone-maintained therapy, 15 (15.96%) were on buprenorphine, and 67 (71.28%) received no substitution therapy. Duration of abstinence was shorter in patients receiving maintenance therapy (mean ± SD: 3.61 ± 2.33 vs. 5.41 ± 3.26 y, P = 0.0170). To determine the influence of substitution therapy on treatment course, SVR and lost to follow-up rates, were compared in IVDUs receiving substitution therapy and those who did not receive substitution therapy. The treatment completion rate was better in the former group. However, patients on substitution therapy had lower SVR rates measured by intention-to-treat analysis, mainly because of a higher rate of lost to follow-up found in those who received substitution therapy (Table 4).

**Table 4:** Influence of Substitution Therapy on the Level of Adherence to Treatment

	**Methadone/Buprenorphine Therapy (n = 27)**	**No Substitution Therapy (n = 67)**	**P value b**
SVR (ITT analysis a), No. (%)	15 (55.55)	50 (74.62)	0.0866
SVR genotype 1 and 4	5 (38.46)	32 (72.72)	0.0442
SVR genotype 2 and 3	10 (71.42)	18 (78.26)	0.7046
Lost of follow-up, No. (%)	7 (25,92)	7 (10.44)	0.1050
Treatment completion rate, No. (%)	27 (100)	64 (95.52) c	0.5546

^a^ Abbreviation: ITT, intention-to-treat analysis

^b^ Fisher's exact test

^c^ In three patients treatment was discontinued

### 4.5. Predictors of SVR

Univariate and multivariate binary logistic regression analyses were performed to identify independent predictors of SVR (Table 5). Univariate analysis revealed five significant associations: Patients' age (< 40 years), genotypes 2 and 3, extent of liver fibrosis (mild fibrosis: ISHAK 1-2), duration of infection and risk factor IVDU. Patients' weight and type of PEG-IFN-α were not associated with SVR in our model. However, using a multivariate logistic regression model (stepwise backward method) we came out to two indepent predictors of SVR-patients' age < 40 years and genotypes 2 and 3. The area under the receiver-operating-characteristic curve in the fully adjusted binary logistic regression model (with the selected predictors) was 0.718, which indicates a good discriminatory accuracy.

**Table 5:** positive predictors of Sustained Virological Response (SVR)

	**Univariate Analysis**		**Multivariate Analysis^b^**	
	**Crude Odds-Ratio (95% CI^a^)**	**P value**	**Adjusted Odds-Ratio (95% CI)**	**P value**
Patients^c^	2.15 (0.97 – 4.78)	< 0.0001	2.98 (1.50 – 5.84)	0.0017
Genotype 2 and 3	17.36 (4.09 – 73.62)	< 0.0001	25.40 (3.44 – 194.82)	0.0016
Mild fibrosis (ISHAK score 0,1,2)	1.87 (0.79 – 4.42)	0.0149	-	-
Duration of infection	0.96 (0.93 – 0.99)	0.0265	-	-
Intravenous drug use (risk factor)	3.38 (1.17 – 9.73)	0.0236	-	-

^a^ Abbreviation: CI, confidence interval

^b^ Complete data for 172 patients

^c^ Age < 40 y

## 5. Discussion

According to the World Health Organization, the estimated prevalence of HCV infection in general population in Croatia is 1.4%, while in IVDUs it is up to 69% [16][18][20]. IVDUs represent significant part of the population treated at Croatian Reference Center for Viral Hepatitis, as 30.46% of treated patients in study period were previous IVDUs. Many patients with CHC who are active or past IVDUs are still excluded from treatment programs. However, results from our study confirm previously published data which shows that IVDUs can be treated effectively and safely [13][15][21]. Also, our study showed that IVDUs have even higher SVR rates, most likely because they have positive predictors for achieving SVR such as younger age, shorter duration of infection, and lower fibrosis score.

Currently, in Western Europe and the USA the genotype 1b is strongly associated with transfusion-related HCV infection, while HCV in IVUDs is mainly associated with genotypes 1a and 3a [4][22][23]. Genotype distribution among IVDUs in our study follows the previously reported pattern. Additionally, the genotype distribution in this study in comparison with previously published data from our center shows an increasing trend in frequency of genotypes 1a and 1b in IVDUs [24]. Similarly, authors from France reported a significant increase in prevalence of genotypes 1a and 3a in IVDUs during the last three decades [25].

One of the main concerns when treating IVDUs is their adherence to treatment. The reported completion rate among IVDUs is around 70%, with significant differences among countries and medical centers [12]. Treatment completion rate in our study was 97.17% in IVDUs, and 92.89% in non-IVDU group. Additionally, treatment completion rate was better in patients receiving substitution therapy than in those who did not, although we noticed a higher rate of patients who were lost to follow-up in patients who received substitution therapy. Patients were closely monitored during the treatment and coming for regular controls every four weeks in our center, and if they experienced any new side-effects they could contact us at any time. Additional follow-up was provided by psychiatrist at centers for treatment of addiction. This could explain the very good adherence rate observed in IVDUs which was even higher than non-IVDU group. It has been shown that substitution therapy effectively reduces the opiate need, HIV risk behaviors and transmission, general mortality and criminal behavior [26][27]. Methadone maintenance treatment (MMT) programs, which require regular contact with patients, presumably promote adherence to antiviral treatment. There are several studies that promote positive effects of MMT therapy and show that comprehensive, on site model of drug abuse treatment with CHC management has improved the SVR rate [14][21][26][27]. Meanwhile, IVDUs treated at specialized liver clinics without integral care model reported lower adherence and SVR rates [11][12][15].

Results from a recently published meta-analysis have shown that tolerability of CHC treatment in IVDUs is comparable to that of general population, with rate of psychiatric side-effects being 2% [15]. Our study also showed that treatment with PEG-INF-α and RBV is safe in IVDUs, but with close psychiatric monitoring. Safety and tolerability profiles in IVDU and non-IVDU group were similar, with psychiatric side-effects (anxiety, depression and insomnia) were more frequent in IVDU group, although not at a statistically significant level. An interesting point of our study is that, although there was no statistically significant difference in treatment modification or discontinuation between two studied groups, a higher trend toward discontinuation was noticed in non-IVDU group than in IVDU group (7.11% vs. 2.83%, P = 0.139). In an Italian study discontinuation rate due to severe depression was 3.8%; in our study only 1.8% of IVDUs had to discontinue treatment for severe depression [14].

Recent studies suggested that IVDUs treated with PEG-IFN-α and RBV can achieve an SVR rate equivalent to that obtained in registration trials, with SVR rates ranging from 39% to 61% [12][15][28]. Differences in SVR rates could be explained by different study design and inclusion of active IVDUs. In our study, SVR rate for IVDUs was 65.1% for genotypes 1 and 4, and 79.1% for genotypes 2 and 3, which was higher than that in non-IVDU group (48.7% and 69.1%, respectively). Results of this study were somewhat similar to the study of Manolakopoulos, et al, where SVR rates were also higher than those in registration trials, with an SVR rate for genotypes 1 and 4 being 57.6%, and for genotypes 2 and 3 was 78.3%. Higher SVR rates in IVDU group can be explained by significantly higher proportion of patients who were young, had shorter duration of infection, lower fibrosis score and higher percentage of genotypes 2 and 3 [21]. The limitations of this study included its retrospective nature, collection of data from a single institution, and a modest sample size. Although we present data from a single institution, our hospital is the Croatian Reference Center for Viral Hepatitis, where the majority of patients from all Croatia are treated. It was beyond the scope of this study to review every chart to determine what percentage of all IVDUs patients with CHC were evaluated for and offered HCV treatment. Interestingly, the patients with longer abstinence period and who did not receive substitution therapy usually came to seek the treatment on their own decision, while patients receiving substitution therapy were recommended for the treatment by their psychiatrists. Our data clearly suggest that adherence of IVDUs was excellent which resulted in a high SVR rate. Treatment of CHC in IVDUs should strongly be encouraged as they present a group with favorable predictors for achieving SVR. Timing for therapy for patients with substitution therapy and adherence, as well as treatment of side effects, should be considered as an important goal of multidisciplinary approach. On the other hand, the awareness of successful treatment of HCV infection can be an important "turning" point in reducing or stopping the substance abuse.
